# Sex and Gender Aspects in Vestibular Disorders: Current Knowledge and Emerging Perspectives—A Systematic Review

**DOI:** 10.3390/diagnostics16020197

**Published:** 2026-01-08

**Authors:** Leonardo Franz, Andrea Frosolini, Daniela Parrino, Giulio Badin, Chiara Pavone, Roberta Cenedese, Agnese Vitturi, Margherita Terenzani, Charles Nicholas Babb, Cosimo de Filippis, Elisabetta Zanoletti, Gino Marioni

**Affiliations:** 1 Phoniatrics and Audiology Unit, Department of Neuroscience DNS, University of Padova, AULSS 2 Ospedale Ca’ Foncello, 31100 Treviso, Italy; leonardo.franz@unipd.it (L.F.); robertacenedese@gmail.com (R.C.); cosimo.defilippis@unipd.it (C.d.F.); 2 Maxillofacial Surgery Unit, Department of Medical Biotechnology, S. Maria alle Scotte University Hospital of Siena, 53100 Siena, Italy; andreafrosolini@gmail.com; 3 Department of Otorhinolaryngology Head and Neck Surgery, ASST Sette Laghi, Ospedale di Circolo e Fondazione Macchi, 21100 Varese, Italy; daniela.parrino@gmail.com; 4 Otolaryngology Unit, Department of Neuroscience DNS, University of Padova, Azienda Ospedale—Università Padova, 35100 Padova, Italy; badingiulio@gmail.com (G.B.); agnese.vitturi@studenti.unipd.it (A.V.); elisabetta.zanoletti@unipd.it (E.Z.); 5 Guided Therapeutics (GTx) Program, Techna Institute, University Health Network, Toronto, ON M5G 2C4, Canada; 6 Otolaryngology Unit, Department of Neuroscience DNS, University of Padova, AULSS 2 Ospedale Ca’ Foncello, 31100 Treviso, Italy; chiara.pavone@aulss2.veneto.it; 7 AULSS 6 Euganea, 35131 Piove di Sacco, Italy; margherita.terenzani@gmail.com; 8 Otolaryngology Associates of Tennessee, Nashville, TN 37209, USA

**Keywords:** gender medicine, sex, vestibular disorders, vertigo, balance

## Abstract

**Background/Objectives**: As precision medicine advances, attention to sex and gender determinants across epidemiological and clinical domains has intensified. However, in the audio-vestibular field, knowledge on sex- and gender-related aspects remains relatively limited. The main aim of this review has been to analyze the available gender medicine-based evidence in vestibular disorders. In particular, our investigation considered the following: (i) pathophysiology and clinical presentation, including differences in predominant signs and symptoms, diagnostic modalities and findings, underlying biological mechanisms associated with vestibular disorders across sex-specific groups; (ii) prognostic variables, including response to treatment, recovery rates, and long-term functional outcomes; (iii) the potential role of sex- and gender-specific diagnostic and therapeutic approaches in the management of vestibular disorders. **Methods**: Our protocol was registered on PROSPERO (CRD42025641292). A literature search was conducted screening PubMed, Scopus and Web of Science databases. After removal of duplicates and implementation of our inclusion/exclusion criteria, 67 included studies were identified and analyzed. **Results**: Several studies reported a higher incidence of vestibular dysfunctions among females, with proposed associations involving hormonal fluctuations, calcium metabolism and vitamin D. Estrogen receptors within the inner ear and their regulatory effects on calcium homeostasis have been proposed as potential mechanisms underlying these sex-specific differences. Furthermore, lifestyle factors, comorbidities and differential health-seeking behaviors between males and females may also modulate disease expression and clinical course. **Conclusions**: Gender-specific variables could not be independently analyzed because none of the included studies systematically reported gender-related data, representing a limitation of the available evidence. Current evidence suggests the presence of sex-related differences in the epidemiology and clinical expression of vestibular disorders, but substantial gaps remain regarding mechanisms, outcomes, and clinical implications. Future research should prioritize prospective, adequately powered studies specifically designed to assess sex and gender influences, integrating biological, psychosocial, and patient-reported outcomes, and adopting standardized sex- and gender-sensitive reporting frameworks.

## 1. Introduction

The emerging interest in precision medicine approaches has led to an increasing attention on differences in prevalence and clinical presentation with respect to gender and biological sex [1]. In several clinical fields, the limited availability of sex and gender-balanced evidence has made research on this topic particularly compelling. In clinical conditions in which a strong male or female prevalence exists, the skewed distribution of cases has often led to disregarding possible clinical differences in patients from the less prevalent gender [2].

In the scientific literature, the terms sex and gender have been distinctly employed to point to different concepts. While sex refers to the biological attributes (such as genetic composition, endocrine profiles, tissue-specific responses, and reproductive anatomy) which typically classify individuals as female or male, gender encompasses the social and cultural constructs that shape roles, identities, behaviors, and relations within a given society [3]. As a result, sex-based differences often underlie fundamental biological mechanisms, while gender-based differences may influence health-seeking behaviors, treatment adherence, and access to care [4].

In response to this, gender medicine, a form of precision medicine, has emerged as a novel clinical paradigm, allowing for a more comprehensive evaluation of the influence of sex and gender on many conditions in terms of risk factors, prognosis, clinical signs, pathophysiology, epidemiology, and therapy [5]. Despite these advances, knowledge of sex- and gender-related aspects in the audio-vestibular field remains relatively limited [6]. Although the information is rather heterogeneous, an overall female epidemiological prevalence in vestibular disorders has been reported for many conditions [7,8]. The recognition of such differences is crucial to the development of tailored treatment approaches for vestibular disorders.

The main aim of this systematic review was to collect and analyze the available sex and gender evidence in vestibular disorders. In particular, the investigation has considered: (i) pathophysiology and clinical presentation, including differences in signs and symptoms, diagnostic findings, and underlying biological mechanisms; (ii) prognostic variables, including response to treatment, recovery rates, and long-term functional outcomes; (iii) the potential role of sex- and gender-specific therapeutic approaches in the management of vestibular disorders.

## 2. Materials and Methods

### 2.1. Protocol Registration

The protocol of this review was registered on PROSPERO, an international database of prospectively registered systematic reviews in health and social care (Center for Reviews and Dissemination, University of York, York, UK) on 19 February 2025 (registry number CRD42025641292).

### 2.2. Definitions

For the purposes of this review, ‘sex’ was defined as a biological attribute in accordance with Sex and Gender Equity in Research (SAGER) Guidelines [9]. ‘Gender’, defined as a sociocultural identity, was not independently studied as gender data were not consistently reported in the vestibular literature. However, the term ‘gender’ was used in our search query to capture articles that may have used sex- and gender-related terminology interchangeably or with alternative definitions.

### 2.3. Search Strategy

A systematic literature review was conducted according to the Preferred Reporting Items for Systematic Reviews and Meta-Analyses (PRISMA) recommendations [10]. (see Supplementary Materials, too). The electronic databases Scopus, PubMed and Web of Science were searched, considering a time frame from 2000 to 2025, according to the following keywords: “gender medicine” OR (gender OR sex) AND (distribution OR difference) AND (vertigo OR benign paroxysmal positional vertigo OR vestibular migraine OR vestibular neuritis OR Ménière’s disease). The reference lists of all the included articles were screened for further relevant studies.

After duplicates removal, 3 reviewers (GB, AV, MT) independently screened all titles and abstracts and then evaluated the full texts of the eligible articles based on the inclusion criteria. Any disagreement between these reviewers was resolved through discussion with all authors to reach a consensus.

### 2.4. Selection Criteria, Data Extraction and Quality Assessment

The retrieved articles were included in the systematic review based on the following inclusion criteria: (i) original reports (including prospective or retrospective studies, with or without control groups); (ii) availability of epidemiological, clinical, or outcomes data with respect to sex or gender; (iii) English publication language. The exclusion criteria were: (i) lack of relevant data; (ii) non-original studies (i.e., reviews, recommendations, letters, editorials); (iii) publication date earlier than 1 January 2000; (iv) case reports and small case series (fewer than 10 subjects), in order to reduce small-sample bias and improve the interpretability of sex-stratified analyses.

Once the included studies were defined, the extracted data together with study characteristics were collected in an electronic database. Furthermore, the quality of the studies eligible for inclusion was independently assessed by three reviewers (GB, AV, MT) and categorized as Poor, Fair, and Good, in agreement with the National Institutes of Health (NIH) quality assessment tool for Observational Cohorts and Cross-Sectional Studies (https://www.nhlbi.nih.gov/health-topics/study-quality-assessment-tools, accessed on 20 July 2025). Any disagreements were resolved by consensus through discussion.

### 2.5. Qualitative and Quantitative Synthesis

The included investigations were qualitatively synthetized. Quantitative synthesis was not feasible for other domains, including diagnostic findings, treatment response, and prognostic outcomes, due to heterogeneity in outcome definitions, measurement tools, and reporting formats, as well as the frequent absence of sex-stratified data. Because cohorts and settings were expected to vary, we prespecified a random-effects model using restricted maximum likelihood (REML) to estimate the pooled mean difference and between-study variance (τ^2^). In view of the anticipated heterogeneity, any quantitative synthesis was considered exploratory and hypothesis-generating rather than confirmatory. Statistical heterogeneity was quantified with Cochran’s Q, I^2^ and H^2^. Two-sided α = 0.05 was used for significance testing. Analyses were conducted with the Meta suite of STATA 16.1 (Stata Corp LP, College Station, TX, USA).

## 3. Results

### 3.1. Search Results, Quality Assessment and Study Design

**Figure 1:**
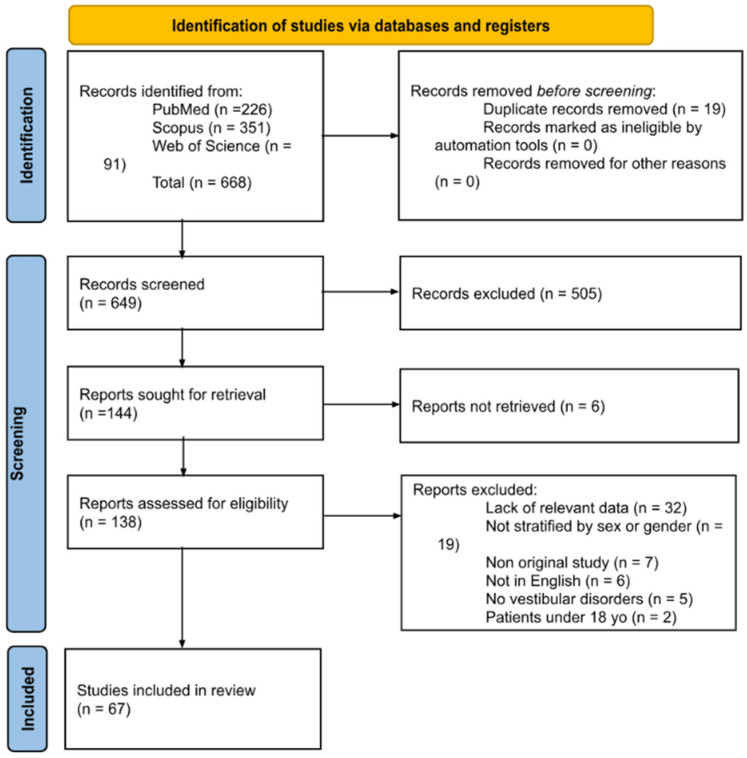
PRISMA diagram summarizing Electronic Database Search and Inclusion/Exclusion process of the review (date of last search 17 February 2025).

A total of 668 papers were retrieved from the literature search. After duplicate removal, records were screened, full-text reports were assessed for eligibility, and 67 studies were included in the review (Figure 1). A list of full-text articles assessed for eligibility but excluded, with reasons, is provided in Supplementary Material S1 in accordance with PRISMA 2020.

The 67 included studies were analyzed for design, quality, population, vestibular disorder type, outcomes and main conclusions [11,12,13,14,15,16,17,18,19,20,21,22,23,24,25,26,27,28,29,30,31,32,33,34,35,36,37,38,39,40,41,42,43,44,45,46,47,48,49,50,51,52,53,54,55,56,57,58,59,60,61,62,63,64,65,66,67,68,69,70,71,72,73,74,75,76,77]. A total of eleven studies were prospective cohort studies [13,16,22,27,33,41,42,52,53,67,72]. Thirty articles employed a retrospective cohort design [12,20,21,25,26,31,32,37,40,44,45,46,47,48,49,50,55,56,58,60,61,63,64,66,69,70,74,75,76,77]. Three studies used a case–control design [27,59,71]. In addition, ten papers were cross-sectional studies [14,17,18,23,26,38,62,65,68,73]. One study was a randomized controlled trial [24], two were non-randomized controlled trials [19,51], and one was a non-controlled clinical trial [29]. Finally, eight papers were population-based studies [11,17,34,35,36,43,44,57]. Twenty-two out of 67 considered a multicentric setting [11,13,17,18,21,34,35,36,38,39,40,41,43,44,48,55,56,57,61,68,73,76]. In agreement with the National Institutes of Health (NIH), the quality assessment analysis reported good quality for 27 papers, fair quality for 38 papers, poor quality for 2 papers (Supplementary Material S1).

Eleven articles included in this review explicitly aimed to highlight potential sex differences in the considered series [13,19,30,34,42,46,47,49,63,72,74]. Among the 67 studies included in this review, 53 studies [11,12,13,14,16,17,18,19,20,21,22,23,24,25,26,27,28,29,30,31,32,34,35,36,37,38,39,40,41,42,43,44,45,46,47,48,49,50,51,52,53,54,55,56,57,58,59,60,61,62,63,64,65,66,67,68,69,70,71,72,73,74,75,76,77] reported significant sex-related differences regarding epidemiology, diagnostic and therapeutic approaches, risk factors, comorbidities and clinical presentation. Conversely, 14 studies [15,20,22,24,27,29,50,54,59,65,66,69,70,74] did not report significant differences. None of the studies included in the review explicitly applied or referenced the SAGER guidelines [78].

### 3.2. Population, Epidemiology and Demographic Characteristics

All of the included articles reported a stratification of demographics and/or clinical characteristics by sex. In 9 studies, the exact vestibular diagnosis was not available [13,14,26,38,43,45,57,65,69], while the remaining 58 reported data about the vestibular conditions affecting their populations. Specifically, 34 included cases of benign paroxysmal positional vertigo (BPPV) [11,12,13,15,16,17,18,20,21,22,24,25,27,29,30,32,34,35,36,37,38,42,46,47,48,49,50,52,53,59,62,63,66,70,71,72,73,75,76], 25 Ménière’s disease (MD) [11,12,16,17,19,25,26,33,34,35,36,37,38,40,42,48,55,56,60,61,62,70,71,73,76], 5 vestibular migraine (VM) [26,28,37,40,62], 11 vestibular neuritis (VN) [34,35,36,37,40,60,61,70,71,72,73,75,76], and 5 persistent postural-perceptual dizziness (PPPD) [15,20,31,37,77]. Most of these studies included cases of more than one vestibular condition.

Table 1 reports the 18 studies investigating the epidemiology and demographic characteristics of vestibular disorders with possible sex-specific differences [15,17,34,35,37,38,43,55,56,60,61,63,65,67,68,73,74,75].

### 3.3. Diagnostic Approaches, Therapeutic Interventions and Outcomes

As shown in Table 2, sex-related differences in diagnostic approaches for vestibular disorders were investigated by six studies [11,23,33,50,54,72]. Four studies [11,23,33,72] reported significant differences between male and female patients, whereas two [50,54] did not.

As summarized in Table 3, nine studies investigated treatment strategies for vestibular disorders [19,24,27,28,29,40,51,53,58]. Six studies [19,28,40,51,53,58] reported significant differences between female and male patients, whereas three [24,27,29] did not report any significant differences.

As illustrated in Table 4, 34 studies investigated sex-related differences [12,13,14,16,18,20,21,22,25,26,30,31,32,36,39,41,42,44,45,46,47,48,49,52,57,59,62,64,66,69,70,71,76,77]. Among them, 28 studies [12,13,14,16,18,21,25,26,30,31,32,36,39,41,42,44,45,46,47,48,49,52,57,62,64,71,76,77] reported significant differences between males and females, while six studies [20,22,59,66,69,70] found no significant differences.

### 3.4. Exploratory Quantitative Synthesis

Among the assessed domains, only dizziness-related quality of life measured using the Dizziness Handicap Inventory (DHI) was reported in a sufficiently homogeneous and sex-stratified manner to allow quantitative synthesis. Fourteen articles [15,18,24,28,29,31,41,45,50,51,52,53,69,77] included in this review used the DHI to assess patients’ quality of life. Two of those [52,53] did not report DHI scores; one [28] reported only the difference in DHI scores between study groups; and seven [15,24,31,41,50,69,77] did not stratify DHI values by sex or gender. Four studies [18,29,45,51], including a total of 323 female and 140 male participants with vestibular disorders (PC-BPPV, unilateral peripheral vestibular dysfunction, long-COVID dizziness), reported DHI details and were eligible for an exploratory quantitative synthesis. As represented in Supplementary Material S1, the pooled analysis revealed a negligible overall mean difference of −0.13 points (95% CI −8.00 to 7.75; z = −0.03; *p* = 0.97), indicating no consistent sex-related difference in perceived dizziness handicap across studies. However, between-study heterogeneity was substantial (τ^2^ = 53.77; I^2^ = 92.2%; H^2^ = 12.82), with a significant Q test (Q(3) = 20.87; *p* < 0.001), limiting the interpretability of the pooled estimate.

## 4. Discussion

Vestibular disorders may exhibit sex-specific variations due to differences in hormonal regulation, inner ear anatomy and neurophysiological processing between males and females [78,79]. Psychosocial, cultural and economic factors can also contribute to potential gender differences [80,81].

In this regard, the overall certainty of evidence across key outcome domains reported in this systematic review can be considered low to moderate based on the NIH quality assessment. While many epidemiological studies report a higher prevalence of vestibular disorders among females [34,35,38,43,68], some population-based and clinical investigations did not identify significant sex-related differences in prevalence, clinical presentation, or patient-reported outcomes, underscoring the heterogeneity of available evidence [15,65,74]. Moreover, epidemiological sex differences do not in themselves imply causal biological mechanisms; therefore, mechanistic interpretations discussed below—predominantly derived from retrospective or cross-sectional designs with heterogeneous methodologies—should be regarded as biologically plausible hypotheses rather than established explanations. These aspects will be addressed in the subsequent sections and in view of the different vestibular disorders considered in this review.

### 4.1. The Role of Hormones in Vestibular Disorders

Recent reviews have comprehensively highlighted the critical role of sex hormones—specifically estrogen and progesterone—in modulating vestibular function and symptom severity in several conditions [78,79]. The rapid decline in estrogen levels during menopause may correlate with impaired otoconial metabolism, contributing to an increased risk of benign paroxysmal positional vertigo (BPPV) in post-menopausal women [72,82,83,84]. These hypotheses originate from mechanistic and epidemiological studies [84], but also from analyses on animal models [72]. Furthermore, hormonal imbalances have been associated with symptom exacerbation in Ménière’s disease (MD), where estrogen-related changes in inner ear microcirculation might play a role [85]. Estrogen replacement might be beneficial in reducing vestibular symptoms in post-menopausal MD patients [85]. These findings underscore the influence of gonadal hormones on vestibular disorders and point to the potential benefits of incorporating hormonal evaluations into routine clinical assessments [79]. Moreover, emerging evidence suggested that the differences extend beyond hormonal regulation alone [8]. Epidemiological data supported this hypothesis. Hülse et al. (2019) analyzed a sample of over 70 million individuals and found that women were disproportionately affected by vestibular disorders such as BPPV, MD and vestibular migraine (VM) in comparison to men, with the sex difference remaining significant even after menopause [34]. Vereeck et al. (2008) also found that, beside age, sex significantly influenced postural control and balance performance [86]. Analyzing 318 participants (180 women, 138 men), they observed that women exhibited significantly poorer results than men in the Romberg test with eyes closed, standing on one leg with eyes closed, and parallel stance on foam with eyes closed [86]. Anatomical and neuro-chemical variations between male and female vestibular systems provided reasonable explanations for the different manifestations of vestibular dysfunction in women. Moriyama et al. (2007 and 2016) highlighted that females tended to have fewer myelinated axons in the vestibular nerve compared to males, which might influence vestibular signal transmission [87,88]. Additionally, considering an animal model, Ayyildiz et al. (2008) reported differences in size and structure of vestibular nuclei, with reduced volumes in female rats [89]. Neuro-chemical systems, including serotonin, dopamine and GABA, which differ between males and females, may influence vestibular processing by modulating conscious perception, anxiety responses to vestibular symptoms and sensitivity to medications like antihistamines and benzodiazepines, which are commonly prescribed for vestibular disorders [8].

### 4.2. Ménière’s Disease

MD has increasingly been examined through the prism of sex and gender medicine, as the emerging literature suggests potential differences in epidemiology, clinical presentation and therapeutic response between males and females. Several studies have highlighted the female predominance of MD [17,35,50,84,86,89]. A large population-based study in the United Kingdom on 5508 MD patients reported that 65.4% of cases occurred in women, with a mean age at diagnosis of approximately 55 years [17,90,91,92]. Similar findings emerged from an Italian nationwide survey, where 72% of the 520 enrolled patients were females [93]. Analogous epidemiological trends were observed in Japan, with a consistently higher prevalence in females compared to males over several decades [51]. Additionally, in a nationwide Korean large cohort of MD patients from 2008 to 2020, the annual incidence rose from 12.4 per 100,000 persons in 2008 to 50.5 per 100,000 in 2020, with females showing significantly higher prevalence rates than males (70.6% in 2008 and 68.8% in 2020, respectively) [35]. Sex-stratified analyses revealed that in females the incidence was significantly higher in spring and autumn and significantly lower in winter than in summer, whereas males only demonstrated a winter reduction without significant increases in spring or autumn [36].

Seasonal variation in MD might be related to weather conditions, as high humidity and low atmospheric pressure—common in Korean summers—have been linked to vertigo, tinnitus and ear fullness [7,94]. Pressure changes could influence endolymphatic hydrops, while greater physical activity and fatigue in spring through autumn could further trigger symptoms, particularly in males and younger patients [7,94].

Beyond incidence, differences in symptoms and disease burden have also been described. Women frequently report greater symptom severity, including more intense vertigo and higher levels of psychosocial distress, particularly anxiety and depression, compared to men [78]. Moreover, fluctuations in symptom expression have been associated with hormonally sensitive periods, such as the menstrual cycle, pregnancy and menopause, suggesting a potential hormonal modulation of endolymphatic hydrops or fluid homeostasis in the inner ear [33,42,78].

In a comparative study on stress hormones across inner ear disorders, Horner and Cazals [33] found that MD patients showed altered stress-hormone regulation, including a distinctive positive correlation between cortisol and prolactin in females, a pattern absent in males and in other inner ear disorders. This sex-specific coupling supported the hypothesis that prolactin might play a prominent role in symptom expression of female MD patients, potentially contributing to both the observed female predominance and the seasonal vulnerability to environmental and physiological stressors [33]. Furthermore, menstrual-cycle phases could influence MD responses, with some women experiencing post-menstruation symptom reduction, while others with premenstrual magnification patterns showed no changes [42].

An Italian pilot non-randomized controlled study [19] investigated the impact of combined oral contraceptives containing drospirenone and ethinyl estradiol, associated with vestibular rehabilitation therapy, in women with MD who experienced premenstrual exacerbation of symptoms. The combined treatment significantly reduced the frequency and severity of vertigo, tinnitus and hearing fluctuations during the luteal phase [19]. These preliminary findings suggested that drospirenone’s anti-mineralcorticoid and hormonal regulatory effects might help modulate inner ear fluid regulation/balance, and drospirenone-containing contraceptives might represent a promising therapeutic option for managing hormonally influenced fluctuations in MD.

The association with migraine is also worth mentioning. Epidemiological studies have long noted a higher prevalence of migraine among MD patients than in the general population, raising the possibility of shared pathophysiological mechanisms [8,88,89,95]. In a cohort of 95 individuals with unilateral intractable MD, Diao et al. [25] reported that patients with comorbid migraine were more frequently female (72%), experienced a longer disease duration and higher vertigo attack frequency, yet paradoxically showed better residual hearing compared to MD patients without migraine. Interestingly, anatomical variations were also noted, with the migraine subgroup patients displaying poor mastoid pneumatization and reduced sigmoid sinus–external ear canal distance. These findings might indicate a migraine-associated MD subtype, potentially driven by overlapping vascular, neurogenic and inflammatory pathways [96], and the recognition of this phenotype could improve diagnostic precision and guide individualized management, particularly in women, who appear disproportionately affected when migraine and MD coexist [97].

These insights collectively highlight the importance of incorporating a gender-sensitive perspective into MD, as considering hormonal status or stress-related factors in clinical assessment may help targeted interventions and could improve symptom management and guide future research.

### 4.3. Benign Paroxysmal Positional Vertigo

BPPV is the most common form of vertigo, with an annual incidence rate of 10.7–600.0/100,000, accounting for 20–30% of vestibular diagnoses [98]. Like other vestibular disorders, BPPV affects women more frequently than men [7,8,34]: several studies showed a statistically significant female predominance in BPPV [7,12,36,63]. According to the studies by Taura, Yang, and Wang, the prevalence among females was approximately twice than in males (2:1 ratio) [60,67,72]. Additionally, in 8611 patients with BPPV, Adams et al. [10] found that women and patients over 70 years of age were significantly more likely to be diagnosed compared to men and people aged 66 to 69.

As previously stated, sex hormones, particularly estrogen and progesterone, are now widely recognized as crucial in modulating vestibular function and symptom severity in various conditions [78,79]. Moreover, estradiol (E2) is essential for bone growth and the development and maintenance of bone health in adulthood. E2 is involved in the prevention of bone loss by changing the natural regulators of bone mass and maintaining the production of osteoprotegerin. The decline of estrogen levels decreases the rate of bone turnover and consequently causes bone loss. E2 levels are known to decline with age, with a marked decline during menopause. This is potentially relevant for BPPV pathophysiology, since the calcium–vitamin D metabolism is known to affect the stability of the otoconia within the semicircular canals’ ampullae. During the menopausal period, as well as in late pregnancy epochs, fluctuating estrogen levels may be associated with otoconial degeneration, which represents the pathophysiological base for the development of BPPV [47,99].

Additionally, in females, it has been recognized that with increasing age, serum levels of 25-hydroxyvitamin D (25(OH)D_3_) and estradiol (E2) decreased [72]. Yang et al. [72] evaluated 102 postmenopausal women, 52 with BPPV and 50 controls, and highlighted that serum E2 and vitamin 25(OH)D_3_ levels were significantly lower in patients with BPPV than in age-matched controls. This suggests that E2 and 25(OH)D_3_ deficiency may be an important risk factor for idiopathic BPPV in postmenopausal female patients. A large survey by Ogun et al. [47] confirmed the role of menopause, showing that 48.1% of BPPV women experienced their first episode after menopause. The study by Li et al. [39] on 484,303 participants aged 40 to 69 years recruited using the UK Bank proved that elderly females (aged ≥ 60 years old) with osteoporosis had a higher risk of developing BPPV.

Insufficient and lower serum 25(OH)D_3_ was also reported to be associated with BPPV relapse, but without a significant difference in gender-based risk distribution [23,41]. However, Otsuka [49], considering 53 out of 357 with recurrent BPPV, found that the incidence of relapse was higher in females than in males, with 69.8% females versus 30.2% males at the first recurrence, 81.8% versus 18.2%, and 100% versus 0% at the second and third recurrences, respectively. Maintaining adequate levels of vitamin D could help to reduce the risk of otolith-related dizziness and improve the clinical situation in those who suffer from it. Vitamin supplementation should therefore be considered as an adjuvant, as it is easily available and accessible; however, prospective studies are still lacking to confirm whether it can prevent or reduce relapses [41].

Regarding non-pharmacological treatments, the Epley maneuver was reported to be effective and safe for posterior canal BPPV (PC-BPPV), although female gender, sleep disorders and inner ear diseases were associated with recurrence. Analyzing 243 patients with PSC-BPPV, Su et al. [58] found that after canalith repositioning with the Epley maneuver, the recurrence was significantly higher in females than in males. Considering 234 patients with PC-BPPV, Domínguez-Durán et al. [27] reported no significant gender-related differences in recovery, defined as loss of nystagmus regardless of loss of symptoms, 7 days after the same Epley maneuver. The absence of gender difference could be related to residual disability, as demonstrated by Petri’s study, which found an early higher DHI score with severe and moderate handicaps both in women and men with BPPV, that substantially resolved one month after treatment [51]. Also, Carrillo Muñoz patients diagnosed with BPPV-PC in primary care perceived their condition as disabling based on DHI scores, with women reporting higher levels of handicap than males [18]. In 132 BPPV patients, Martens [41] concluded that a higher dizziness handicap was associated with the female gender.

The diagnosis of BPPV is based on the medical history, associated symptoms and the presence of nystagmus; Choi et al. [22], by a questionnaire evaluating the directionality of linear vertigo, found that female gender did not represent an independent risk factor for variations in the clinical presentation of vertigo in BPPV. Regarding the medical history, we should not forget to consider some disorders which, as we found in this review, were more frequent in women with BPPV. Female and younger age (<60 years) with a history of BPPV had a higher likelihood of concurrent comorbidities of BPPV and migraine [33]. Furthermore, in BPPV, metabolic syndrome was more prevalent in females [71]. Female gender was also an independent risk factor for developing BPPV in patients with anxiety disorder, as reported by Chen [21] and Ferrari [30]. In the analysis of some psychological comorbidities and especially their relationship with the handicap reported through self-assessment questionnaires in a sample of patients with dizziness, a greater disability was found in females [21,30,51,52].

The association of BPPV and MD has been reported [100,101]. The percentage of patients with MD experiencing BPPV ranges between 10% and 70% [102,103]. Several authors described that patients with BPPV associated with MD differed from patients with idiopathic BPPV in terms of epidemiological and clinical features [12,101,103,104,105]. Apart from a longer duration of symptoms and a higher recurrence rate [12,101,103,104], a significant female predominance was observed, with an even higher percentage (93%) of female patients affected when the two disorders co-exist [12,101,103,105]. This consistent gender imbalance suggests that women may have increased susceptibility to otoconial detachment in the context of endolymphatic hydrops, potentially mediated by hormonal influences, bone metabolism differences, or inner ear microstructural vulnerability. These findings underscore the need for sex-specific considerations in both diagnostic evaluation and therapeutic strategies for BPPV in MD patients.

### 4.4. Vestibular Migraine

The association between vertigo and migraine, and vice versa, has been widely described, and nowadays it is well established, with VM being a recognized disorder diagnosed according to the criteria defined by the Consensus of Bárány Society [106] and the Third Headache Classification Committee of the International Headache Society [107]. However, the female preponderance of migrainous vertigo was reported even before the proposal of diagnostic criteria for VM [108], with a reported female-to-male ratio ranging between 1.5 and 5 to 1 [109,110,111,112]. The female predominance was confirmed by two large epidemiological studies reporting 75.8% and 81% of women among 584 and 2515 patients affected by VM, respectively [37,113]. Also, the more recent study by Lin et al. (2024) showed a significant female preponderance of VM, with a female-to-male ratio of approximately 3.3:1 [40]. Notably, the female predominance fluctuated with age, being 2.2:1 in patients < 30 years, 4.2:1 in those aged 31–60 years, and 2.9:1 in patients > 60 years. Hormonal influences, particularly fluctuations in estrogen levels and Calcitonin Gene-Related Peptide release, affect the vestibular and trigeminal systems and imply that women are more likely to develop migraine and related vestibular symptoms across reproductive years [114,115]. VM symptoms may worsen due to the hormonal shifts that occur during the menstrual cycle, particularly the drop in estrogen levels in the days leading up to and during menstruation [116,117]. Also, pregnancy can influence vestibular disorders [118], and VM may either improve during gestation due to loss of cyclical fluctuation of estrogen levels or exacerbate and occur ex novo towards the end of pregnancy and early postpartum period, possibly influenced by sleep deprivation, stress and hormonal fluctuations [119].

Evidence for male and female differences in VM treatment response is limited but suggestive. In their series of VM patients primarily treated by trigger management alone, Lin et al. (2024) observed a comparable success rate between males and females by the end of the first-year treatment course, both being greater than 70% [40]. Selectively for females, they observed that women aged > 45 years showed a significantly better improvement from trigger management than younger women. This better control of VM observed in older females, presumably peri- or postmenopausal, may arise from the concomitant stabilization of their reproductive hormone status after menopause [40]. A single-center cohort study [28] found female sex to be a positive predictor of better response to standard VM therapies (i.e., antidepressants, antiepileptics, beta blockers, vestibular rehabilitation); women on average had greater reductions in DHI compared to men, particularly in the emotional and functional domains. The evident sexual dimorphism in VM prevalence may reflect hormonal fluctuations that explain the better response in females [28]. This dimorphism may also represent a gender disparity in reporting VM symptoms, especially non-specific vestibular complaints, as men seem to be more likely to report abnormal anxiety or depression in comparison to women [120]. The previously mentioned finding that women improve more than men suggests that biological sex might be considered when counseling patients about prognosis in VM.

### 4.5. Vestibular Neuritis

VN is traditionally considered a benign, self-limited peripheral vestibular disorder, yet emerging perspectives in gender medicine suggest that sex-related biological and socio-cultural determinants may influence its incidence, clinical expression and outcomes. In many studies [38,60,65,69], which analyzed the epidemiology of vertigo, although VN cases were grouped with other conditions, the sex imbalance suggests potential gender-related vulnerability or healthcare-seeking behavior differences. The incidence of VN was significantly different by sex, age and residence, with the highest values in females, people aged 60 years or older, and people who resided in metropolitan cities. VN is thought to be caused by reactivation of latent virus infection, autoimmunity, or microvascular ischemia [36]. Furthermore, VN could induce anxiety, because it appears with acute severe vestibular symptoms. The higher incidence of VN in women can be related to anxiety, which is more common in females [36]. However, considering the many possible causes of VN, the higher incidence of VN in females cannot be explained by anxiety alone. Hülse et al. emphasized that VN accounted for a notable fraction of acute vestibular presentations and 62.3% were women [34].

Beyond incidence, gender differences may emerge in seasonality and environmental susceptibility. Jeong et al. (2024) demonstrated that the risk of VN in males was lower in autumn and winter than in summer; conversely, in females, the risk of VN was lower in winter than in summer but higher in spring and autumn than in summer [36]. While VN is classically linked to viral etiologies, sex-specific immunological responses may modulate susceptibility and recovery. Future research examining whether hormonal status or gender-related occupational exposures contribute to seasonality would be valuable.

A crucial dimension in gender medicine is symptom perception and healthcare utilization. Petri et al. (2017) assessed quality of life and disability in acute vestibular disorders and found significant impacts on daily functioning [51]. In this study, the most frequent diagnosis in both genders was VN, without significant differences between the two sexes. The study highlighted how the higher improvement in functional, emotional and physical pre-treatment DHI scores at one month after treatment was for patients with vestibular neuritis, a well-treatable condition. In their series, Wilhelmsen et al. (2009) found that sex significantly predicted dizziness and autonomic/anxiety-related symptoms, underlining how the classification of patients was crucial to provide a better basis for specific rehabilitation [70]. In patients with VN, recovery from acute vertigo is within days/weeks. However, residual balance problems are not unusual, because the neuro-otologic condition may have started a process triggering anxiety.

### 4.6. Limitations and Strengths

This review has several limitations. First, the available evidence on sex- and gender-related differences in vestibular disorders remains heterogeneous, as most studies are retrospective, single-center, and lack standardized diagnostic or therapeutic protocols. Sample sizes are often limited, and information on hormonal status is rarely documented or controlled for, limiting the ability to draw robust conclusions about sex-related biological mechanisms. Furthermore, the absence of standardized sex- and gender-sensitive reporting reduces data comparability and precludes comprehensive meta-analytic synthesis. Accordingly, the exploratory quantitative synthesis performed in this review was characterized by substantial statistical heterogeneity and should be interpreted with caution. Finally, the inclusion of multiple vestibular disorders with distinct pathophysiological mechanisms may limit the generalizability of cross-condition comparisons. It should also be noted that the literature is predominantly focused on BPPV and Ménière’s disease, reflecting the distribution of available studies. Recognizing this imbalance is important when interpreting the findings, as the overrepresentation of these conditions might limit the generalizability of our conclusions to less-studied vestibular disorders, such as vestibular migraine, vestibular neuritis, and PPPD.

Despite its limitations, this systematic review comprehensively highlights, in the context of gender medicine, how biological sex- and gender-related factors exert an influence on disease presentation, prevalence and outcomes of many vestibular disorders. Several studies reported a higher incidence of vestibular dysfunctions among women, suggesting that hormonal fluctuations, calcium metabolism, and vitamin D deficiency may contribute to this predisposition. Estrogen receptors within the inner ear and their regulatory effects on calcium homeostasis have been proposed as potential mechanisms underlying these sex-specific differences. Furthermore, lifestyle factors, comorbidities, and differential health-seeking behaviors between men and women may also modulate disease expression and clinical course. These findings underscore the importance of integrating a gender-based approach into the assessment and management of vestibular disorders, to achieve more accurate diagnosis, targeted prevention and individualized treatment strategies.

## 5. Conclusions

From a clinical perspective, the findings of this review suggest that sex- and gender-related factors may represent relevant modifiers of disease risk and clinical expression in vestibular disorders. Although sex- and gender-specific treatment algorithms cannot yet be recommended, awareness of factors such as hormonal status, menopausal transition, and alterations in calcium and vitamin D metabolism may help refine diagnostic assessment and patient counseling. Incorporating molecular and hormonal pathways that underlie sex differences in vestibular dysfunction with psychometric assessments and quality-of-life measures may contribute to disentangling the biological and psychosocial factors influencing disease onset, progression and treatment response.

Future research should prioritize prospective, adequately powered studies specifically designed to assess sex and gender influences in vestibular disorders.

Finally, adopting standardized frameworks for sex/gender data collection and reporting—such as the SAGER guidelines—would improve reproducibility and support a more equitable and personalized approach to vestibular medicine.

## Figures and Tables

**Table 1:** Studies focusing on the epidemiology and demographic characteristics of vestibular disorder.

First Author, Year	Country	Aims of the Study	Study Design	Cases/Condition Studied (N)	Controls (N)	Outcome	Measures	Are There Any Statistically Significant Sex and Gender Differences?
Nakashima T, 2000 [43]	Japan	To investigate the number of patients and clinical manifestations of idiopathic sudden sensorineural hearing loss.	PBS	vertigo NOS (2346)	NA	Epidemiology	Prevalence	YES
Shojaku H, 2005 [55]	Japan	To identify epidemiological characteristics of definite cases of MD.	RS	MD (375)	NA	Epidemiology	Prevalence	YES
Shojaku H, 2009 [56]	Japan	To analyze changes over time in the epidemiological characteristics of MD in Japan.	RS	MD (1368)	NA	Epidemiology, clinical presentation,	Prevalence	YES
Yin M, 2009 [75]	Japan	To investigate the clinical and epidemiological characteristics of vertigo.	RS	BPPV (149), VN (108), MD (96), vestibular dysfunction (20), DEH (19), labyrinth concussion (5), Hunt’s syndrome (5), SSCDS (3), acoustic tumor (137)	NA	Epidemiology, etiology	BVE	YES
Taura A, 2010 [60]	Japan	To investigate the epidemiological features of patients with vertigo.	RS	MD (184), BPPV (149), VN (20), DEH (13), acoustic tumor (8), chronic otitis media (4), perilymphatic fistula (3), large vestibular aqueduct (2)	NA	Epidemiology, clinical presentation,	Frenzel camera, MRI, PTA, DPOAEs	YES
Lai YT, 2011 [38]	Taiwan	To investigate the epidemiology of vertigo among the general adult population in Taiwan using the National Health Insurance claims database.	CSS	Vertigo NOS (527,807)	NA	Epidemiology, risk factors	Prevalence, recurrence	YES
Wahat, 2013 [65]	Malaysia	To assess the prevalence of vestibular disorders in an Otology Service, ENT clinic of UKMMC, Malaysia as an early establishment of epidemiological data of vestibular disorders in Malaysia.	CSS	Vertigo NOS (146)	Other symptoms (631)	Epidemiology, clinical presentation	Clinical assessment, prevalence	NO
Ward BK, 2013 [68]	USA	To assess prevalence and functional impact of BVH in the U.S. adult population.	CSS	Bilateral Vestibular Hypofunction (21,782)	NA	Epidemiology	Clinical evaluation, questionnaire	YES
Wang J, 2014 [67]	China	To explore the prevalence of BPPV in vertigo patients and the characteristics of BPPV in diagnosis and repositioning using mechanical assistance maneuvers.	PS	BPPV (209)	NA	Epidemiology, Pathophysiology	TRV armchair test	YES
Bittar RS, 2015 [15]	Brazil	To evaluate the clinical characteristics of patients with persistent postural and perceptual dizziness.	PCS	PPPD (74)	No chronification (7)	Clinical presentation; comorbidities	DHI; STAI; BDI; HADS	NO
Yetiser S, 2015 [74]	Turkey	To document the demographic data of patients with BPPV regarding distribution of gender, age, associated problems, most common form, symptom duration, severity of nystagmus and cure rate.	RS	BPPV (263)	NA	Epidemiology, Clinical presentation	VNG	NO
Bruderer SG, 2017 [17]	USA	To assess the incidence rates of MD and describe the characteristics of MD cases, comparing them to control patients without recorded evidence of MD.	CCS	MD (5508)	Non MD (22,032)	Epidemiology; clinical presentation; comorbidities, co-medications	Prevalence	YES
Tutar B, 2018 [63]	Turkey	To investigate age, gender, side and type of canal involvement, association with other diseases, response to treatment, and recurrence rate in patients with BPPV.	RS	BPPV (516)	NA	Epidemiology, pathophysiology,	BVE	YES
Hülse R, 2019 [34]	Germany	To undertake a representative epidemiological survey that examines all age groups of an entire population and describes the age and gender distribution of the most common peripheral vestibular disorders.	PBS	Unspecific Vertigo and Dizziness (3,406,169), BPPV (322,164), MD (143,885), VN (114,163)	NA	Epidemiology	Prevalence	YES
Kim HJ, 2020 [37]	South Korea	To determine the etiological distribution of dizziness and vertigo in a referral-based dizziness clinic in South Korea.	RS	BPPV (5943), PPPD (5116), VM (2515), MD (1782), VN (1328), bilateral vestibulopathy (343)	NA	Epidemiology, etiology	Prevalence	YES
Yang TH, 2021 [73]	Taiwan	To evaluate the prevalence of peripheral vestibular disorders in an Asian population.	CSS	other PVD (32,960), BPPV (7321), VN (5038), MD (1154),	NA	Epidemiology	BVE	YES
Taybeh E, 2023 [61]	Jordan	To investigate the profile of hospital admissions linked to inner ear diseases in England and Wales.	RS	BPPV & VN & MD (NA)	NA	Epidemiology	Prevalence	YES
Jeong J, 2023 [35]	South Korea	To investigate the incidence of peripheral vestibular disorders using population-based data representing the whole population of South Korea.	PBS	BPPV (205,011), VN (43,834), MD (32,353)	NA	Epidemiology	Prevalence	YES

Abbreviations: BDI: Beck Depression Inventory; BPPV: Benign Paroxysmal Positional Vertigo; BVE: Bedside Vestibular Examination; CCS: case–control study; CSS: cross sectional study; DEH: delayed endolymphatic hydrops; DHI: Dizziness Handicap Inventory; DPOAEs: Distortion Product Otoacoustic Emissions; HADS: Hospital Anxiety and Depression Scale; MD: Ménière’s Disease; MRI: Magnetic Resonance Imaging; NA: not applicable; NOS: not otherwise specified; PBS: population-based study; PPPD: Persistent Postural-Perceptual Dizziness; PS: prospective study; PTA: pure tone average; PVD: Peripheral vestibular disorder; RS: retrospective study; SSCDS: Superior Semicircular Canal Dehiscence Syndrome; STAI: State-Trait Anxiety Inventory; VN: vestibular neuritis; VNG: Videonystagmography.

**Table 2:** Diagnostic approaches and findings in vestibular disorders.

First Author, Year	Country	Aims of the Study	Study Design	Cases/Condition Studied (N)	Controls (N)	Outcome Measures	Are There Any Statistically Significant Sex and Gender Differences?
Horner KC, 2005 [33]	France	To determine the expression pattern of stress-related hormones in patients with Ménière’s disease or acoustic neuroma, compared to control subjects.	PS	MD (51)	acoustic neuroma; facial spasm (73)	GH, prolactin, cortisol, ACTH serum levels, BVE	YES
Pelosi S,2013 [50]	USA	To characterize patients with isolated unilateral utricular dysfunction with oVEMPs abnormalities and compare them with dizzy patients showing normal BFT results.	RS	unilateral utricular dysfunction (31)	dizziness symptoms with normal BFT results (30)	oVEMP, Balance Function Test, DHI, Hospital Anxiety and Depression Test, prevalence	NO
Adams ME, 2017 [11]	USA	To determine patterns of use of vestibular testing and diagnosis codes for dizziness and balance disorders among individuals over 65 years of age in the United States.	PBS	Dizziness NOS (53,890), BPPV (8611), other PVD (8000), MD (1831)	NA	BVE, Caloric Test; Rotary Chair Test	YES
Yang H, 2018 [72]	China	To provide direct data showing the distinct relationship between female sex hormone fluctuations and BPPV in postmenopausal female patients.	PS	Post-menopausal BPPV (50)	Post-menopausal patients without BPPV (52)	BVE	YES
Cobb LH, 2023 [23]	USA	To examine the relationship of 25(OH)D_3_ serum levels with BPPV incidence and recurrence rates.	CSS	BPPV (173)	healthy subjects (5962)	BVE; 25(OH)D_3_ serology	YES
Shin H, 2023 [54]	Korea Republic	To examine the relationship between serum vitamin D levels and BPPV recurrence.	RS	BPPV recurrence (19)	patients without BPPV recurrence (31)	clinical history questionnaire, auditory function test, cVEMPs, oVEMPs and laboratory tests (including vitamin D level)	NO

Abbreviations: ACTH: Adrenocorticotropic Hormone; BFT: Balance Function Test; BPPV: Benign Paroxysmal Positional Vertigo; BVE: Bedside Vestibular Examination; CSS: cross sectional study; cVEMPs: vestibular evoked myogenic potentials; DHI: Dizziness Handicap Inventory; GH: Growth Hormone; MD: Menière’s Disease; NA: Non-Applicable; NOS: Not Otherwise Specified; oVEMPs: ocular vestibular evoked myogenic potentials; PBS: population-based study; PS: prospective study; PVD: Peripheral vestibular disorder; RS: retrospective study.

**Table 3:** Therapeutic interventions.

First Author, Year	Country	Aims of the Study	Study Design	Condition Studied/Cases/Intervention Group (N)	Controls/Control Group (N)	Outcome Measures	Are There any Statistically Significant Sex and Gender Differences?
Cohen HS, 2003 [24]	USA	To determine the effectiveness in decreasing vertigo and increasing performance of daily life skills after VR.	RCT	Chronic vertigo— Slow head movements while seated (NR)	Chronic vertigo— rapid head movements while seated and while standing /rapid head movements plus attention (NR)	Rotatory and bithermal caloric tests of the VOR in darkness, DHI, VADL	NO
Su P, 2016 [58]	Taiwan	To determine the relationship between variable factors and the recurrence rate of PSC-BPPV after CRP.	RS	PSC-BPPV Recurrence (41)	PSC-BPPV Non-recurrence (202)	BVE	YES
Domínguez-Durán E, 2017 [27]	Germany	To identify risk factors, which predict the Epley maneuver (EM) failure, among the clinical variables recorded in anamnesis and patient examination.	PS	BPPV (234)	NA	BVE	NO
Petri M, 2017 [51]	Brazil	To compare the health-related quality of life of individuals with vestibular disorders of peripheral origin by analyzing functional, emotional and physical disabilities before and after vestibular treatment.	NRCT	acute unilateral vestibular peripheral disorders before treatment (100)	acute unilateral vestibular peripheral disorders after treatment (100)	Health survey on quality of life (SF-36), DHI	YES
Caruso S, 2018 [19]	Italy	To evaluate the effects of a 20 mm EE/3 mg DRSP oral contraceptive in continuous regimen, associated with rehabilitation therapy on MD.	NRCT	MD— Oral contraceptive + rehabilitation (16)	MD— Rehabilitation therapy alone (20)	stabilometry test	YES
Elbeltagy R, 2018 [29]	USA	To determine the efficacy of VR exercise of patients with uncompensated unilateral peripheral vestibular dysfunction.	UCT	unilateral peripheral vestibular dysfunction (20)	NA	DHI	NO
Dornhoffer JR, 2021 [26]	Germany	To identify patient factors that influence response to therapy in patients with vestibular migraines.	RS	VM— Botulinum toxin injections + Rehabilitation protocol (5)	VM— Rehabilitation protocol alone (42)	DHI; BVE	YES
Plescia F, 2021 [53]	Italy	To evaluate efficacy of the fixed combination when used to reduce symptoms of vestibular vertigo of central and/or peripheral origin, after both the 15- and 60-day therapies.	PS	Vertigo NOS (120)	NA	Visual Scale of Dizziness Disorders, DHI	YES
Lin KY, 2024 [40]	USA	To describe the demographic features of VM compared to other common peripheral vestibulopathies, and to assess the efficacy of trigger management as primary VM treatment.	RS	VM (204), MD (150), BPPV (110), VN (97)	NA	Self-reported symptoms; BVE	YES

Abbreviations: 25(OH)D_3_: 25-hydroxyvitamin D; BPPV: Benign Paroxysmal Positional Vertigo; BVE: Bedside Vestibular Examination; CRP: canalith repositioning procedure; DHI: Dizziness Handicap Inventory; DRSP: drospirenone; EE: etinilestradiol; EM: Epley maneuver; MD: Menière’s Disease; NA: Not Applicable; NOS: Not Otherwise Specified; NR: not reported; NRCT: non-randomized controlled trial; PS: prospective study; PSC: Posterior Semicircular Canal; RCT: randomized controlled trial; RS: retrospective study; UCT: uncontrolled trial; VADL: Vestibular Disorders Activities of Daily Living Scale; VM: Vestibular Migraine; VN: Vestibular Neuritis; VOR: Vestibulo-Ocular Reflex; VR: Vestibular Rehabilitation.

**Table 4:** Symptoms, comorbidities, prognosis and quality of life outcomes.

First Author, Year	Country	Aims of the Study	Study Design	Cases/Condition Studied (N)	Controls (N)	Outcome	Measures	Are There Any Statistically Significant Sex and Gender Differences?
Baré M, 2023 [13]	Spain	To assess potential sex-based differences among older patients hospitalized for chronic disease exacerbation.	PS	Vertigo NOS (740)	NA	Epidemiology	Prevalence	YES
Balatsouras DG, 2012 [12]	Greece	To identify demographic, pathogenetic and clinical features of BPPV associated with MD.	RS	BPPV + MD (29)	idiopathic BPPV (138)	Epidemiology, clinical features	Duration of symptoms; Vertigo severity; VNG	YES
Bisdorff A, 2013 [14]	France	To explore the prevalence, demographic and clinical characteristics, and comorbidities associated with vertigo, dizziness, and unsteadiness in a representative sample of the French population.	CSS	Vertigo, dizziness, unsteadiness (2987)	NA	Epidemiology; clinical presentation; comorbidities	VSS; Marks fear questionnaire; MSSQ	YES
Brantber K, 2011 [16]	USA	To investigate whether clinical features of vertigo attacks can distinguish patients with BRV (benign recurrent vertigo) from those with Menière’s disease and whether subtypes of BRV can be identified.	PS	BRV (63)	MD (112)	Clinical presentation	Vertigo attack duration; Time to recover after an attack	YES
Carrillo Muñoz R, 2019 [18]	Spain	To describe how patients diagnosed with posterior canal BPPV in primary care perceive disability.	CSS	BPPV (134)	NA	Clinical presentation; comorbidities	DHI-S	YES
Casani AP, 2023 [20]	Italy	To identify the predictive clinical elements of evolution towards PPPD.	RS	s-PPPD (51)	BPPV and no evolution to PPPD (107)	Risk factors, clinical presentation	Caloric testing, vHIT, VEMPs	NO
Chen ZJ, 2016 [21]	Taipei	To evaluate the risk of BPPV among patients with anxiety disorders by using the Taiwan National Health Insurance Research Database (NHIRD).	RS	BPPV + anxiety disorders (178)	BPPV alone (71)	Risk factors, clinical presentation, comorbidities	Prevalence, comorbidities	YES
Choi JY, 2021 [22]	South Korea	To compare the differences in clinical presentation and nystagmus characteristics between patients with PC-BPPV and those with HC-BPPV.	PS	posterior canal BPPV (63)	horizontal canal BPPV (36)	Clinical presentation, pathophysiology,	BVE, subjective questionnaire	NO
Diao T, 2022 [25]	China	To explore the clinical and anatomical features in MD patients with and without migraine, determining whether the coexistence of migraine can be used as a basis for clinical subtyping of MD.	RS	MD (95)	healthy controls (95)	Clinical presentation, comorbidities	BVE	YES
Dlugaiczyk J, 2021 [26]	Swiss	To characterize the clinical picture of RVS-NOS and to compare it to MD and VM.	CSS	VM (150), MD (119), RVS-NOS (35)	NA	Clinical presentation, comorbidities	PEVS questionnaire	YES
Ferrari S, 2014 [30]	Italy	To evaluate psychiatric-psychosomatic comorbidities in a group of BPPV patients.	CCS	BPPV (92)	healthy controls (141)	Risk factors, clinical presentation	BDI, STAI, DCPR, BSI, and TAS-20	YES
Habs M, 2020 [31]	Germany	To describe typical demographic and clinical features in p-PPPD and s-PPPD patients.	RS	p-PPPD (195)	s-PPPD (162)	Clinical presentation, comorbidities	Length of vertigo episodes, DHI, vHIT gain, posturography	YES
Hilton DB, 2020 [32]	USA	To investigate whether the presence or absence of migraine is associated with increased prevalence of other factors in the largest reported U.S. cohort of BPPV patients.	RS	BPPV (1481)	NA	Clinical presentation, comorbidities	Prevalence	YES
Jeong J, 2024 [36]	South Korea	To investigate seasonal variation in peripheral vestibular disorders using data.	PBS	BPPV, VN, MD (NR)	NA	Risk factors, pathophysiology, epidemiology	Incidence	YES
Li J, 2023 [39]	UK	To analyze the relationship between low BMD and the risk of BPPV based on the large prospective population-based UK Biobank cohort.	PBS	BPPV (985)	NA	Risk Factors	TDI	YES
Martens C, 2019 [41]	Norway	To assess dizziness handicap and clinical characteristics of posterior and lateral canal BPPV.	PS	BPPV (132)	NA	Clinical presentation	DHI; VNG; 25-hydroxyvitamin D-level.	YES
Morse GG, 2001 [42]	USA	To establish the relationship between menstrual cycle phases and MD responses.	PS	MD menstruant women (13)	MD men (12)	Risk factors, clinical presentation	Subjective questionnaires	YES
Neuhauser H, 2005 [44]	Germany	To determine the prevalence and incidence of vestibular vertigo in the general population and to describe its clinical characteristics and associated factors.	PBS	vestibular vertigo (243)	NA	Epidemiology, risk factors	Prevalence, recorded symptoms	YES
Obeidat FS, 2023 [45]	Jordan	To explore the presence and correlates of dizziness and hearing loss in a sample of people with long-COVID syndrome.	RS	Long COVID with dizziness/hearing loss (209)	NA	Clinical presentation	Subjective questionnaires, tympanometry, PTA	YES
Ogun OA, 2014 (A) [46]	USA	To examine the age and gender distribution and the effect of menopause in a large cohort of participants diagnosed with BPPV.	RS	BPPV (1377)	NA	Epidemiology, risk factors	Subjective questionnaire, BVE	YES
Ogun OA, 2014 (B) [47]	USA	To investigate comorbidities that predispose an individual to freeing otoconia and not location of otoconia displacement in BPPV patients.	RS	BPPV (227)	no-BPPV (1360)	Comorbidities, risk factors, epidemiology	Subjective questionnaire, BVE	YES
Onuki J, 2005 [48]	Japan	To investigate the possibility that daily lifestyle may have a causal relationship with MD.	RS	MD (179)	Idiopathic low frequencies hearing loss without vestibular disorders (140)	Risk factors	PTA, Bekesy test, glycerol test, BVE, subjectives questionnaires	YES
Otsuka K, 2013 [49]	Japan	To examine the clinical features, age and gender distribution of patients, treatment methods, and outcomes of benign paroxysmal positional vertigo.	RS	BPPV (357)	NA	Clinical presentation	BVE	YES
Piker EG, 2008 [52]	USA	To evaluate the distribution of anxiety, depression, somatic awareness, autonomic symptoms and differences in coping strategies in patients with vestibular disorders.	PS	BPPV (63)	NA	Clinical presentation	DHI, Vertigo Symptoms Scale (VSS), HADS, Ways of Coping Questionnaire	YES
Skoien AK, 2008 [57]	Norway	To assess the incidence of dizziness/vertigo in long-term sickness absence and to identify sociodemographic and diagnostic predictors for transition into disability pension.	PBS	vertigo NOS (1020)	NA	Epidemiology, clinical presentation, prognostic variables, associated disability	Prevalence	YES
Tan J, 2017 [59]	China	To determine the clinical characteristics and outcomes of h-BPPV, as well as the clinical differences between h-BPPV and i-BPPV.	CCS	h-BPPV (41)	i-BPPV (47)	Epidemiology, Recurrence rate, prognostic factors	BVE	NO
Toupet M, 2019 [62]	France	To investigate the relation between visual and vestibular hypersensitivity, and Depersonalization/Derealization symptoms in patients with chronic dizziness.	CSS	adult patients with chronic dizziness for more than 3 months (319)	NA	Epidemiology, etiology	DDI, HAD, DDA scores	YES
Wada M, 2017 [64]	Japan	To examine associations between smoking and new PVD events.	RS	PVD ever-smokers (109)	PVD never-smokers (284)	Epidemiology, risk factors, prognostic variables	BVE	YES
Waissbluth S, 2023 [66]	Chile	To evaluate the prevalence of cardiovascular risk factors in patients with benign paroxysmal positional vertigo secondary to AUPVP and analyze differences in prior history of BPPV, affected semicircular canals, and response to repositioning maneuvers between patients with idiopathic benign paroxysmal positional vertigo and secondary to acute unilateral peripheral vestibulopathy.	RS	BPPV Secondary to AUPVP (84)	idiopathic BPPV (158)	Epidemiology, pathophysiology	Caloric testing, vHIT	NO
Wassermann A, 2021 [69]	Germany	To define age-specific characteristics of dizziness/vertigo.	RS	chronic vertigo NOS (1752)	NA	Epidemiology, clinical presentation	DHI	NO
Wilhelmsen K, 2009 [70]	Norway	To assess the epidemiological and clinical features of patients experiencing chronic dizziness.	RS	MD (92), BPPV (90), VN (89), Vestibular schwannoma (40)	NA	Epidemiology, prognostic factors, clinical presentation	Vertigo Symptom Scale	NO
Yamanaka T, 2013 [71]	Japan	To determine the prevalence of MetS in vertigo patients and clinically investigated the association between MetS and vertigo.	CCS	MD with MetS (10), BPPV with MetS (7)	MD without MetS (51), BPPV without MetS (43)	Epidemiology, risk factors,	BVE	YES
Zborayova K, 2024 [76]	Sweden	To describe the vertigo/dizziness sick leave prevalence and duration considering both specific and nonspecific diagnoses according to ICD-10.	RS	BPPV & VN & MD & vertigo NOS (45,353)	NA	clinical presentation	Prevalence	YES
Zhang L, 2022 [77]	China	To analyze the differences in the clinical characteristics of PPPD with different patient age groups and different etiologies.	RS	p-PPPD (86), s-PPPD (36)	NA	Epidemiology, clinical presentation, risk factors	BVE, VOR, caloric test, MRI, neck vascular US, transcranial Doppler, blood test, DHI, ABC, beck anxiety inventory, PHQ 9	YES

Abbreviations: ABC: Activities-specific Balance Confidence; AUPVP: Acute Unilateral Peripheral Vestibulopathy; BDI: Beck Depression Inventory; DHI: Dizziness Handicap Inventory; BPPV: Benign Paroxysmal Positional Vertigo; BSI: Brief Symptom Inventory; BVE: Bedside Vestibular Examination; CCS: Case–Control Study; CSS: cross sectional study; DCPR: Diagnostic Criteria for Psychosomatic Research; DDA: Dizziness in Daily Activity; DDI: Depersonalization/Derealization Inventory; DHI: Dizziness Handicap Inventory; HADS: Hospital Anxiety and Depression Scale; h-BPPV: Benign Paroxysmal Positional Vertigo with hypertension; i-BPPV: idiopathic Benign Paroxysmal Positional Vertigo; MD: Menière’s Disease; MetS: Metabolic Syndrome; MRI: Magnetic Resonance Imaging; MSSQ: Motion Sickness Susceptibility Questionnaire; NA: Not Applicable; NOS: Not Otherwise Specified; NR: not reported; PEVS: Prospective study on the phenotype of episodic vestibular syndromes; PHQ-9: Patient health questionnaire 9 items; PPPD: Persistent Postural-Perceptual Dizziness; p-PPPD: Primary Persistent Postural-Perceptual Dizziness; PS: Prospective Study; PBS: Population Based Study; PTA: Pure Tone Average; PVD: Peripheral Vestibular Disorder; RS: Retrospective Study; RVS: Recurrent Vestibular Symptoms; s-PPPD: secondary Persistent Postural-Perceptual Dizziness STAI: State-Trait Anxiety Inventory; TAS-20: Toronto Alexithymia Scale; TDI: Townsend Deprivation Index; v-HIT: video Head Impulse test; VM: Vestibular Migraine; VN: Vestibular Neuritis; VNG: Videonystagmography; VOR: Vestibulo-Ocular Reflex; VSS: Vertigo Symptom Scale.

## Data Availability

The original contributions presented in this study are included in the article/Supplementary Material. Further inquiries can be directed to the corresponding author.

## Supplementary Materials

The following supporting information can be downloaded at: https://www.mdpi.com/article/10.3390/diagnostics16020197/s1, S1: PRISMA Checklist, Excluded studies, Table “Quality Assessment in agreement with the National Institutes of Health quality assessment tool for Observational Cohorts and Cross-Sectional Studies”, Random-effects (REML) forest plot of mean Dizziness Handicap Inventory (DHI) differences (Female—Male).
