# Supplementary material for: Sex and Gender Aspects in Vestibular Disorders: Current Knowledge and Emerging Perspectives—A Systematic Review
Source: Diagnostics (Basel). 2026 Jan 8;16(2):197. doi: 10.3390/diagnostics16020197 (PMC12839779; doi:10.3390/diagnostics16020197)
Supplement: Supplementary file 1 [file diagnostics-16-00197-s001.zip › diagnostics-4061445-supplementary.pdf]

1. Random-effects (REML) forest plot of mean Dizziness Handicap Inventory (DHI) differences (Female − Male).

Positive values indicate higher handicap in females.

Weights are inverse-variance under the random-effects model.

Heterogeneity statistics are shown below the pooled estimate.

This analysis should be interpreted as exploratory and hypothesis-generating rather than confirmatory.

	Female			Male				
Study	N	Mean DHI	SD	N	Mean DHI	SD	Mean DHI difference with 95% CI	Weight (%)
Carrillo Muñoz R.	102	16.83	10.15	32	10	6.21	6.83 [3.91, 9.75]	28.81
Elbeltagy R.	12	52.42	3.29	8	50.22	2.33	2.20 [-0.26, 4.66]	29.14
Obeidat F.S	137	25.7	24.1	72	37.8	30	-12.10 [-20.12, -4.08]	22.88
Petri M.	72	52.17	26.1	28	52	24.22	0.17 [-10.64, 10.98]	19.16
**Overall**							-0.13 [-8.00, 7.75]	

Heterogeneity: $\tau^2 = 53.77$, $I^2 = 92.20\%$, $H^2 = 12.82$

Test of $\theta_i = \theta_j$: $Q(3) = 20.87$, $p = 0.00$

Test of $\theta = 0$: $z = -0.03$, $p = 0.97$

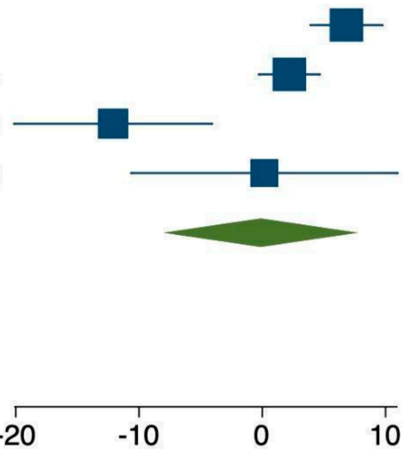

2. PRISMA 2020 Checklist

Section and Topic	Item #	Checklist item	Location where item is reported (page; table and figure)
**TITLE**			
Title	1	Identify the report as a systematic review.	1
**ABSTRACT**			
Abstract	2	See the PRISMA 2020 for Abstracts checklist.	1
**INTRODUCTION**			
Rationale	3	Describe the rationale for the review in the context of existing knowledge.	1
Objectives	4	Provide an explicit statement of the objective(s) or question(s) the review addresses.	1
**METHODS**			
Eligibility criteria	5	Specify the inclusion and exclusion criteria for the review and how studies were grouped for the syntheses.	2
Information sources	6	Specify all databases, registers, websites, organisations, reference lists and other sources searched or consulted to identify studies. Specify the date when each source was last searched or consulted.	2
Search strategy	7	Present the full search strategies for all databases, registers and websites, including any filters and limits used.	2
Selection process	8	Specify the methods used to decide whether a study met the inclusion criteria of the review, including how many reviewers screened each record and each report retrieved, whether they worked independently, and if applicable, details of automation tools used in the process.	2
Data collection process	9	Specify the methods used to collect data from reports, including how many reviewers collected data from each report, whether they worked independently, any processes for obtaining or confirming data from study investigators, and if applicable, details of automation tools used in the process.	2
Data items	10a	List and define all outcomes for which data were sought. Specify whether all results that were compatible with each outcome domain in each study were sought (e.g. for all measures, time points, analyses), and if not, the methods used to decide which results to collect.	2
	10b	List and define all other variables for which data were sought (e.g. participant and intervention characteristics, funding sources). Describe any assumptions made about any missing or unclear information.	2
Study risk of bias assessment	11	Specify the methods used to assess risk of bias in the included studies, including details of the tool(s) used, how many reviewers assessed each study and whether they worked independently, and if applicable, details of automation tools used in the process.	2; Table 1
Effect measures	12	Specify for each outcome the effect measure(s) (e.g. risk ratio, mean difference) used in the synthesis or presentation of results.	2
Synthesis methods	13a	Describe the processes used to decide which studies were eligible for each synthesis (e.g. tabulating the study intervention characteristics and comparing against the planned groups for each synthesis (item #5)).	2
	13b	Describe any methods required to prepare the data for presentation or synthesis, such as handling of missing summary statistics, or data conversions.	2
	13c	Describe any methods used to tabulate or visually display results of individual studies and syntheses.	2; Table 1-5 and Figure

Section and Topic	Item #	Checklist item	Location where item is reported (page; table and figure)
			2
	13d	Describe any methods used to synthesize results and provide a rationale for the choice(s). If meta-analysis was performed, describe the model(s), method(s) to identify the presence and extent of statistical heterogeneity, and software package(s) used.	2; Figure 2
	13e	Describe any methods used to explore possible causes of heterogeneity among study results (e.g. subgroup analysis, meta-regression).	2
	13f	Describe any sensitivity analyses conducted to assess robustness of the synthesized results.	2
Reporting bias assessment	14	Describe any methods used to assess risk of bias due to missing results in a synthesis (arising from reporting biases).	2
Certainty assessment	15	Describe any methods used to assess certainty (or confidence) in the body of evidence for an outcome.	2
**RESULTS**			
Study selection	16a	Describe the results of the search and selection process, from the number of records identified in the search to the number of studies included in the review, ideally using a flow diagram.	3-12
	16b	Cite studies that might appear to meet the inclusion criteria, but which were excluded, and explain why they were excluded.	Herein reported (see below)
Study characteristics	17	Cite each included study and present its characteristics.	3-12
Risk of bias in studies	18	Present assessments of risk of bias for each included study.	3-12
Results of individual studies	19	For all outcomes, present, for each study: (a) summary statistics for each group (where appropriate) and (b) an effect estimate and its precision (e.g. confidence/credible interval), ideally using structured tables or plots.	3-12
Results of syntheses	20a	For each synthesis, briefly summarise the characteristics and risk of bias among contributing studies.	3-12
	20b	Present results of all statistical syntheses conducted. If meta-analysis was done, present for each the summary estimate and its precision (e.g. confidence/credible interval) and measures of statistical heterogeneity. If comparing groups, describe the direction of the effect.	3-12
	20c	Present results of all investigations of possible causes of heterogeneity among study results.	3-12
	20d	Present results of all sensitivity analyses conducted to assess the robustness of the synthesized results.	3-12
Reporting biases	21	Present assessments of risk of bias due to missing results (arising from reporting biases) for each synthesis assessed.	3-12
Certainty of evidence	22	Present assessments of certainty (or confidence) in the body of evidence for each outcome assessed.	3-12
**DISCUSSION**			
Discussion	23a	Provide a general interpretation of the results in the context of other evidence.	13-17
	23b	Discuss any limitations of the evidence included in the review.	13-17

Section and Topic	Item #	Checklist item	Location where item is reported (page; table and figure)
	23c	Discuss any limitations of the review processes used.	13-17
	23d	Discuss implications of the results for practice, policy, and future research.	13-17
**OTHER INFORMATION**			
Registration and protocol	24a	Provide registration information for the review, including register name and registration number, or state that the review was not registered.	2
	24b	Indicate where the review protocol can be accessed, or state that a protocol was not prepared.	2
	24c	Describe and explain any amendments to information provided at registration or in the protocol.	NA
Support	25	Describe sources of financial or non-financial support for the review, and the role of the funders or sponsors in the review.	18
Competing interests	26	Declare any competing interests of review authors.	18
Availability of data, code and other materials	27	Report which of the following are publicly available and where they can be found: template data collection forms; data extracted from included studies; data used for all analyses; analytic code; any other materials used in the review.	18

From: Page MJ, McKenzie JE, Bossuyt PM, Boutron I, Hoffmann TC, Mulrow CD, et al. The PRISMA 2020 statement: an updated guideline for reporting systematic reviews. BMJ 2021;372:n71. doi: 10.1136/bmj.n71.
This work is licensed under CC BY 4.0. To view a copy of this license, visit https://creativecommons.org/licenses/by/4.0/

3. Full-text articles assessed for eligibility but excluded, with reasons

Non-original studies (n = 7)

The following full-text articles were excluded because they were narrative reviews, overviews, or state-of-the-art papers and did not present original clinical data:

- Corazzi (2020)– *Gender differences in audio-vestibular disorders*
- da Costa (2002)– *Ménière's disease: overview, epidemiology, and natural history*
- El Khiati (2023) – *Vestibular Disorders and Hormonal Dysregulations: State of the Art and Clinical Perspectives*
- Furman (2013) – *Vestibular migraine: Clinical aspects and pathophysiology*
- Lempert (2009) – *Vertigo as a Symptom of Migraine*
- Smith (2019) - *Sexual dimorphism in vestibular function and dysfunction*
- Wipperman (2014) - *Dizziness and Vertigo*

Patients under 18 years of age (n = 2)

The following studies were excluded because they exclusively investigated pediatric populations:

- Hülse (2020) – *Prevalence of peripheral vestibular diseases in children in Germany*
- Zhang Q (2022) – *Characteristics of vestibular migraine, probable vestibular migraine, and recurrent vertigo of childhood in caloric and video head impulse tests*

Supplementary Materials of "Gender medicine in vestibular disorders: current knowledge and emerging perspectives. A systematic review"

No vestibular disorders (n = 5)

The following articles were excluded because vestibular disorders, as defined by the review eligibility criteria, were not the primary condition under investigation:

- Anson (2021) - *Association between vestibular function and rotational spatial orientation perception in older adults*
- Chen (2021) - *REM-related obstructive sleep apnea and vertigo: A retrospective case-control study*
- Jones (2022) - *Efficacy and safety of esketamine nasal spray by sex in patients with treatment-resistant depression: findings from short-term randomized, controlled trials*
- Mahulu (2019) - *The variation of superior semicircular canal bone thickness in relation to age and gender*
- Mosallanezhad (2012) - *Physical activity and physical functioning in Swedish and Iranian 75-year-olds - a comparison*

Not stratified by sex or gender (n = 19)

The following studies were excluded because sex or gender was not reported in a stratified or extractable manner for the outcomes of interest:

- Ardç (2006) – *Impact of multiple etiology on dizziness handicap*
- Batuecas-Caletrio (2014) – *The vestibulo-ocular reflex and subjective balance after vestibular schwannoma surgery*
- Bener (2024) - *The Impact of Serums Calcium 25-Hydroxy Vitamin D, Ferritin, Uric Acid, and Sleeping Disorders on Benign Paroxysmal Positional Vertigo Patients*
- Black (2004) – *Benign paroxysmal positional nystagmus in hospitalized subjects receiving ototoxic medications*
- Carmona (2022) - *Atypical Positional Vertigo: Definition, Causes, and Mechanisms*
- Çelebisoy (2022) - *Vestibular migraine, demographic and clinical features of 415 patients: A multicenter study*

- Chihara (2012) – *Clinical characteristics of inferior vestibular neuritis*
- David (2024) - *Vestibular Rehabilitation Using Dynamic Posturography: Objective and Patient-Reported Outcomes from a Randomized Trial*
- Faralli (2014) - *Benign paroxysmal positional vertigo and migraine: analysis of 186 cases*
- Guilemany (2004) – *Clinical and Epidemiological Study of Vertigo at an Outpatient Clinic*
- Hummel (2014) - *Vestibular loss and balance training cause similar changes in human cerebral white matter fractional anisotropy*
- Jeong (2021) – *Monthly and seasonal variations in benign paroxysmal positional vertigo*
- Mikulec (2010) - *Negative association between treated osteoporosis and benign paroxysmal positional vertigo in women*
- Oh (2001) - *Familial benign recurrent vertigo*
- Okuda (2021) - *Differences in responsiveness of intratympanic steroid injection for intractable vertigo in Meniere's disease*
- Plodpai (2014) - *The characteristic differences of benign paroxysmal positional vertigo among the elderly and the younger patients: A 10-year retrospective review*
- Reyhani (2024) - *Comparison of the Degree of Handicap Between Different Types of Vestibular Disorders*
- Shigeno (2006) - *Intractable BPPV*
- Yetişer (2017) - *Co-existence of Benign Paroxysmal Positional Vertigo and Meniere's Syndrome*

Lack of relevant or extractable data (n = 32)

Several full-text articles were excluded because outcomes relevant to the objectives of the review were not reported in a usable or extractable form (e.g., absence of analyzable clinical outcomes, focus on physiological or imaging correlates without clinical correlation, or insufficient reporting). Representative examples include:

Supplementary Materials of “Gender medicine in vestibular disorders: current knowledge and emerging perspectives. A systematic review”

- Aggarwal (2000) - The prevalence of dizziness and its association with functional disability in a biracial community population
- Beh (2019) - *The Spectrum of Vestibular Migraine: Clinical Features, Triggers, and Examination Findings*
- Brantberg (2005) - *Migraine-associated vertigo*
- Ghosh A (2023) - *Epidemiology of benign paroxysomal positional vertigo (BPPV) and risk factors for secondary BPPV: a population-based study*
- Guidetti (2005) - *The recurrences of paroxysmal positional vertigo*
- Jeong (2022) - *Monthly and seasonal variations in vestibular neuritis*
- Jönsson (2004) - *Prevalence of dizziness and vertigo in an urban elderly population*
- Jung (2016) - *Comparison between objective and subjective benign paroxysmal positional vertigo: clinical features and outcomes*
- Kerrigan (2013) -*Prevalence of Benign Paroxysmal Positional Vertigo in the Young Adult Population*
- Kim (2015) - *Effect of hearing loss, age, and gender on the outcome of the cochlear hydrops analysis masking procedure*
- Kısabay (2022) - *Factors determining the response to treatment in patients with vestibular migraine*
- Kollén (2012) - *Benign paroxysmal positional vertigo is a common cause of dizziness and unsteadiness in a large population of 75-year-olds*
- Kovari (2020) - *Comorbidity clusters in generalized osteoarthritis among female patients: A cross-sectional study*
- Kumar (2024) - *Demographic Variations in VEMP Responses: A Cross-Sectional Study of Normative Data from an Indian Population*
- Lee (2012) - *Clinical characteristics and natural course of recurrent vestibulopathy: A long-term follow-up study*
- Li (2021) - *The Impact of Coronavirus Disease 2019 Epidemic on Dizziness/Vertigo Outpatients in a Neurological Clinic in China*
- Muelleman (2017) - *Epidemiology of Dizzy Patient Population in a Neurotology Clinic and Predictors of Peripheral Etiology*
- Neuhauser (2007) - *Epidemiology of vertigo*

Supplementary Materials of "Gender medicine in vestibular disorders: current knowledge and emerging perspectives. A systematic review"

- Neuhauser (2006) - *Migrainous vertigo: prevalence and impact on quality of life*
- Park (2024) - *Clinical characteristics of persistent postural-perceptual dizziness and its visual subtype in Korean patients: A multicenter cross-sectional study*
- Perez-Garrigues (2008) - *Time Course of Episodes of Definitive Vertigo in Meniere's Disease*
- Shami (2011) - *Causes of Vertigo in Saudi patients seen at tertiary teaching hospital*
- Wellons (2024) - *Investigating health disparities in vestibular rehabilitation*
- Patangay (2016) - *Benign Paroxysmal Positional Vertigo: Our Experience*
- Plescia (2021) - *Pharmacological treatment of dizziness*
- Russo (2018) - *Autoimmune vertigo: an update on vestibular disorders associated with autoimmune mechanisms*
- Sinha (2017) - *Menstrual cycle effects on sacculocollic reflex pathway*
- Tan (2018) - *Our experience with 500 patients with benign paroxysmal positional vertigo: Reexploring aetiology and reevaluating MRI investigation*
- Teranishi (2012) - *Polymorphisms in genes involved in oxidative stress response in patients with sudden sensorineural hearing loss and Ménière's disease in a Japanese population*
- Tian (2022) - *A Preliminary Study on the Characteristics and Standard Diagnosis and Treatment of Vestibular Dysfunction in Children*
- Ray (2021) - *Association Between Cardiometabolic Factors and Dizziness in African Americans: The Jackson Heart Study*
- Yetiser (2022) - *Pseudo-spontaneous nystagmus in patients with lateral canal benign paroxysmal positional vertigo*
